# Coordinate aware implicit neural representation for UAV small object detection

**DOI:** 10.1371/journal.pone.0350990

**Published:** 2026-06-10

**Authors:** Yong Yang, Tianci Wan, Ling Guo, Menglu Zhang

**Affiliations:** 1 School of Computer Science, Zhengzhou University of Aeronautics, Zhengzhou, China; 2 Department of Archives, Zhengzhou University of Aeronautics, Zhengzhou, China; Xidian University, CHINA

## Abstract

Small object detection in unmanned aerial vehicle (UAV) imagery remains challenging due to low resolution, complex backgrounds, and limited pixel occupancy. Conventional CNN-based detectors rely on fixed receptive fields, which often fail to capture fine-grained spatial details and implicit geometric relationships. To address these limitations, this paper proposes a Coordinate-Aware Implicit Neural Representation Enhanced C2INR module that integrates coordinate-based representations into convolutional feature extraction. The Multi-Scale Coordinate Encoder (MSCE) constructs frequency-aware positional embeddings through sinusoidal encoding to enhance spatial continuity at multiple scales. The INR Feature Enhancer (IFE) further fuses encoded coordinates with visual features via lightweight MLP modulation, improving sensitivity to small-scale variations. Additionally, a Small Object Attention mechanism combines global context, local detail, and high-frequency cues to strengthen responses to tiny targets. Experiments on three UAV benchmarks—AI-TOD, UAVDT, and VisDrone—demonstrate consistent improvements over existing methods with minimal computational overhead. Further evaluation on PASCAL VOC verifies strong cross-domain generalization. These findings confirm that coordinate-aware implicit representation provides an effective and broadly applicable solution for improving spatial continuity, geometric fidelity, and localization precision in small-object detection.

## 1 Introduction

Unmanned aerial vehicle (UAV) object detection has attracted increasing research interest in recent years due to its wide applications in surveillance, traffic monitoring, and search-and-rescue missions. However, detecting small objects in UAV imagery remains extremely challenging because of limited spatial resolution, large scale variations, and complex backgrounds [1]. Small targets typically occupy only a few pixels, resulting in weak semantic cues and high susceptibility to background clutter. These issues collectively degrade detection robustness, particularly when objects are distant, partially occluded, or embedded in densely textured scenes.

**Fig 1:**
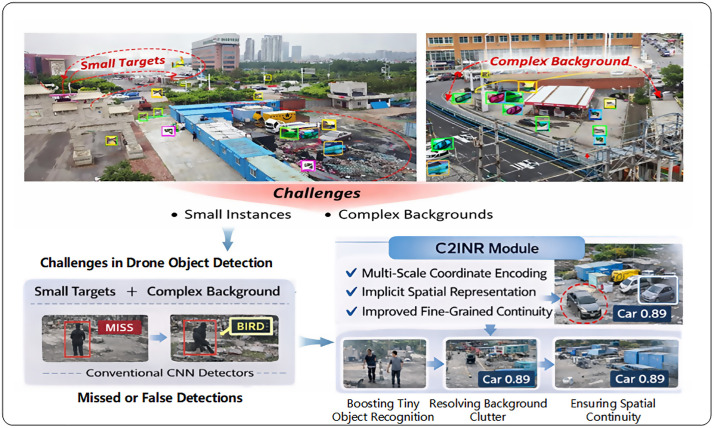
Challenges in UAV small-object detection and the role of the proposed C2INR module.

As illustrated in Fig 1, UAV imagery typically suffers from two representative difficulties: extremely small foreground instances and highly cluttered backgrounds. Under such conditions, conventional CNN-based detectors frequently produce missed detections or false positives, primarily because discrete convolutional sampling lacks sufficient spatial awareness and fine-grained continuity modeling. This observation highlights the necessity of introducing more explicit spatial modeling mechanisms for reliable small-object perception.

Recent studies have advanced UAV small-object detection from various perspectives, including multi-scale feature fusion, frequency-aware representation learning, attention enhancement, and transformer-based design. For instance, SF-DETR introduces a Multi-Scale Frequency Fusion module and dual-scale transformer backbones to jointly exploit spatial and frequency-domain features, achieving superior detection of small objects in dense UAV scenes [2]. MGDFIS enhances both global contextual cues and local detail perception through attention-guided directional convolutions [3], while AUHF-DETR employs wavelet convolutions and adaptive cross-scale decoupling modules to preserve high-frequency texture information [4]. These methods improve feature richness and multi-scale adaptability. However, as visualized in Fig 1, most existing approaches still rely on discrete convolutional or token-based representations that perform static aggregation or attention reweighting without explicitly encoding spatial coordinates or modeling continuous spatial relationships. Consequently, they often fail to maintain fine-grained spatial continuity and precise localization, especially for extremely small or low-contrast targets under complex UAV imaging conditions.

Motivated by the above observations, this work explores whether continuous coordinate-aware modeling can provide more robust spatial sensitivity for UAV small-object detection. Implicit Neural Representation (INR) was originally proposed for modeling continuous signals, geometric surfaces, and 3D shapes. Its core idea is to learn a mapping function $f_{\theta }:{\mathbb{R}}^{n}\to {\mathbb{R}}^{m}$ that transforms continuous coordinates into signal values, thereby achieving spatial resolution-independent feature representation. The Sinusoidal Representation Network (SIREN) proposed by Sitzmann et al. [5] first validated this concept, demonstrating remarkable expressiveness in implicit scene reconstruction and high-frequency signal recovery. Subsequently, Cao et al. [6] extended INR to 3D scene reconstruction by conditionally generating high-fidelity volumetric representations.

In recent years, INR has shown growing potential in vision tasks that demand high-precision spatial modeling or resolution-independent representation. For example, Gu et al. [7] proposed Neural Vector Fields for Implicit Surface Representation and Inference, which models coordinate-conditioned vector fields to capture sub-pixel geometric features for fine structural perception. Similarly, Cao et al. [8] in Visible and Clear: Finding Tiny Objects in Difference Map, employed coordinate-conditioned reconstruction to enhance subtle difference regions, improving small object detection performance. These studies collectively highlight the strong spatial continuity and detailed representational capability of the INR framework.

Despite these advances, integrating the INR paradigm into convolutional neural network (CNN)-based UAV detection remains largely unexplored. Conventional approaches such as multi-scale fusion or attention mechanisms mainly reweight discrete features without explicitly modeling spatial continuity. To bridge this gap, this paper introduces the Coordinate-aware Implicit Neural Representation, which jointly encodes coordinate embeddings and visual features to construct a unified coordinate-semantic representation space. This mechanism enables sub-pixel geometric correction and semantic consistency enhancement on convolutional feature maps, yielding more precise, continuous, and scale-consistent representations. Consequently, coordinate-aware implicit representation establishes a new paradigm for feature modeling in UAV-based small object detection.

To address these challenges, we further develop a Coordinate-Aware Implicit Neural Representation framework tailored for UAV object detection. The main contributions are summarized as follows.

1. A C2INR module integrates coordinate-aware implicit representation into CNN-based detection frameworks, enabling explicit continuous spatial modeling within standard convolutional pipelines.
2. A Multi-Scale Coordinate Encoder (MSCE) is designed using multi-frequency sinusoidal functions with adaptive scale factors to capture fine-grained spatial dependencies across different resolutions.
3. An Implicit Feature Enhancer (IFE) fuses coordinate and visual embeddings through lightweight MLP-based modulation and residual refinement, enhancing feature continuity and localization precision.

## 2 Related work

### 2.1 Small object detection in UAV imagery

Small object detection in UAV imagery has attracted extensive research due to its practical importance and intrinsic challenges such as low target resolution, large viewpoint variation, and severe background clutter. Recent approaches fall into several categories. Multi-scale feature fusion methods aim to alleviate scale mismatch by aggregating features from different layers or by designing specialized neck architectures for denser feature reuse [9]. Transformer-based and attention-enhanced detectors further improve contextual modeling and long-range dependency capture, which are beneficial for small-object discrimination in cluttered scenes [10]. Frequency-aware and wavelet-based methods have also been proposed to preserve high-frequency texture useful for tiny object localization [11]. Despite these advances, maintaining precise localization and spatial continuity for extremely small or low-contrast targets remains challenging, motivating coordinate- or resolution-aware design choices.

### 2.2 Attention, frequency and hybrid methods

Attention mechanisms and frequency-domain processing have been widely adopted to enhance discriminability for small targets. Channel and spatial attention blocks reweight features to emphasize important channels or regions [12]. Frequency-aware modules, including discrete Fourier or wavelet transforms, aim to separate and boost high-frequency components that carry edge and texture cues for tiny objects [13]. Hybrid strategies combine attention with frequency filtering to simultaneously model context and texture [14]. While effective, these methods typically act on discrete feature maps and do not explicitly impose coordinate continuity, which may limit localization accuracy for sub-pixel-scale targets.

### 2.3 Coordinate and positional representations in vision

Explicit coordinate or positional encoding has a long history in vision and sequence models. Classical approaches include learned or sinusoidal position embeddings used in transformers [15] and coordinate convolution variants (CoordConv) that append coordinate channels to convolutional inputs [16]. More recently, continuous coordinate conditioning via implicit neural representations (INRs) has been studied for tasks requiring resolution-agnostic or sub-pixel precision. INR methods such as SIREN and NeRF demonstrate how coordinate-to-signal mappings can recover high-frequency details and continuous fields [17]. Applications of INR-like ideas to image-level tasks (e.g., implicit vector fields, coordinate-conditioned reconstruction) suggest potential benefits for dense prediction and small-object localization. However, direct integration of INR into mainstream CNN detection pipelines is still limited. Despite their effectiveness, existing coordinate-aware strategies exhibit notable limitations. Transformer positional encodings primarily provide additive or concatenative location cues in a discrete token space and remain largely content-agnostic, which restricts their ability to adapt spatial responses to object scale variations. CoordConv improves spatial awareness by explicitly appending normalized coordinate channels, yet it still relies on fixed, hand-crafted spatial representations and lacks the capacity to model continuous coordinate-conditioned feature modulation. In addition, recent frequency-aware attention mechanisms enhance feature selectivity in the spectral domain, but they typically operate on transformed feature responses rather than establishing an explicit continuous mapping between spatial coordinates and semantic features.

In contrast to the above approaches, the proposed C2INR introduces an implicit continuous coordinate-conditioned modulation framework within the detection backbone. Instead of injecting explicit coordinate channels or fixed positional embeddings, C2INR learns a continuous mapping from spatial coordinates to feature recalibration weights, enabling content-adaptive spatial sensitivity. This design allows the network to capture fine-grained spatial variations and improves localization capability for small objects while maintaining compatibility with standard CNN detection pipelines.

## 3 Methods

### 3.1 Overview of the proposed framework

**Fig 2:**
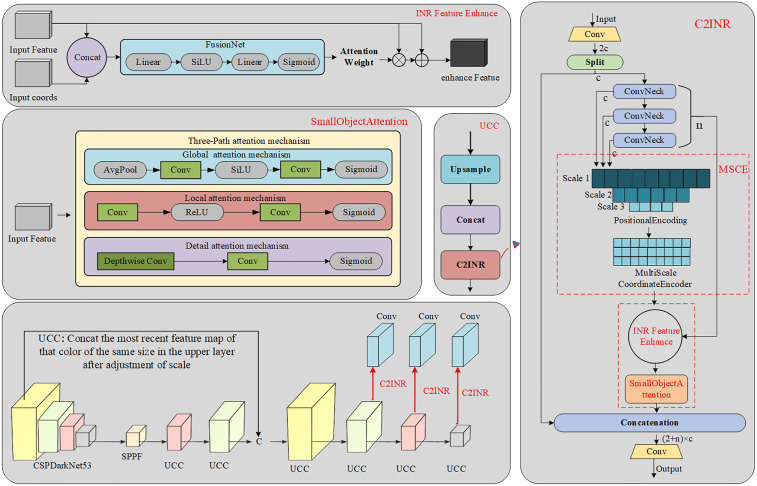
C2INR integrates the Multi-Scale Coordinate Encoder (MSCE) and INR Feature Enhancer (IFE) to achieve coordinate-aware and continuous feature representation. The Small Object Attention module further refines discriminative cues, while UCC connections embed C2INR into the backbone to enhance multi-scale detection performance.

The overall structure of the proposed UAV object detection framework is shown in Fig 2. Built upon the CSPDarkNet53 backbone, it integrates multiple C2INR modules to enhance feature continuity and spatial localization. The CSPDarkNet53 backbone extracts hierarchical visual features, while the UCC (Upsample and Cross-layer Concatenation) mechanism connects feature maps of the same resolution across layers to strengthen multi-scale interaction. The core C2INR module combines convolutional processing with coordinate modeling. Inside C2INR, the MSCE encodes positional information at multiple frequencies, and the IFE fuses coordinate and visual embeddings through lightweight MLP-based modulation. Additionally, the Small Object Attention branch refines global, local, and detail cues to improve small-target perception. By jointly modeling coordinate and semantic representations, the proposed framework achieves continuous, scale-consistent, and fine-grained feature representations, significantly improving UAV small object detection accuracy.

### 3.2 Coordinate aware implicit neural representation (C2INR)

From a functional modeling perspective, UAV small-object detection can be interpreted as learning a spatially conditioned mapping


$$\begin{array}{c} {ℱ}_{\Theta }:(𝐗,𝐩)\to 𝐘, \end{array}$$

where **X** denotes the visual feature field and $𝐩\in {\mathbb{R}}^{2}$ represents continuous spatial coordinates. Conventional CNN-based detectors approximate this mapping primarily through discrete convolutional sampling, which may introduce spatial discontinuities and limit fine-grained localization, especially for small or sparsely represented targets.

To address this limitation, the proposed C2INR explicitly models a coordinate-conditioned continuous function


$$\begin{array}{c} f_{\theta }(𝐩,𝐗), \end{array}$$

which aims to approximate a smooth coordinate-to-feature response field over the spatial domain. By integrating multi-scale coordinate encoding with implicit feature modulation, C2INR preserves the continuous function approximation property of classical INR while remaining compatible with standard CNN detection pipelines. Guided by the above continuous modeling perspective, to embed the proposed coordinate-aware implicit representation into the detection backbone, this study integrates the Multi-Scale Coordinate Encoder (MSCE) and INR Feature Enhancer (IFE) into a unified module termed C2INR. This design follows the structural spirit of the C2f block while introducing implicit coordinate modeling to enhance spatial continuity and fine-grained feature consistency.

In conventional C2f structures, feature refinement is achieved through a series of convolutional bottlenecks that primarily focus on local texture aggregation. However, such discrete operations lack explicit spatial awareness, leading to discontinuities when representing small or distant targets. The C2INR addresses this limitation by injecting coordinate priors and implicit feature modulation into the standard bottleneck flow.

Specifically, given the input feature $X\in {\mathbb{R}}^{B\times C_{1}\times H\times W}$, it is first divided into two parallel branches via a 1 × 1 convolution:


$$\begin{array}{c} {}[y_{0},y_{1}]={\text{Conv}}_{1\times 1}(X). \end{array}$$

The first branch (*y*_0_) serves as the shortcut path, while the second branch (*y*_1_) passes through *n* iterative bottleneck layers, each incorporating MSCE and IFE to progressively refine spatial and semantic representations. Within each bottleneck, MSCE encodes multi-scale coordinate priors, while IFE performs implicit modulation to align coordinate and visual feature spaces, resulting in enhanced spatial continuity.

After iterative enhancement, the outputs from both branches are concatenated and fused through a final 1 × 1 convolution to obtain the output feature:


$$\begin{array}{c} Y_{\text{out}}={\text{Conv}}_{1\times 1}(\text{Concat}(y_{0},y_{1}^{*})), \end{array}$$

where $y_{1}^{*}$ denotes the coordinate-enhanced feature produced by the sequential MSCE–IFE refinement process. This design ensures that each C2f block not only aggregates multi-scale texture information but also maintains geometric consistency across spatial domains.

By embedding coordinate-aware implicit modeling within the C2f architecture, the C2INR module effectively bridges the gap between discrete convolutional representation and continuous spatial reasoning. It enhances the network’s capability to capture small, low-contrast, and scale-varying targets in UAV imagery, thereby improving both localization precision and detection robustness.

#### 3.2.1 Multi-scale coordinate encoder (MSCE).

To enhance the perception of spatial distribution and geometric layout in UAV imagery, this study introduces a MSCE that explicitly models positional information across multiple spatial resolutions. Unlike conventional sinusoidal or learned positional embeddings, which remain fixed after training and fail to adapt to scale variations, MSCE dynamically encodes coordinate priors from both global and local perspectives, enabling the network to construct a continuous coordinate field that is well suited for small-object representation.

The MSCE begins by generating a normalized coordinate map (*x*, *y*) for each spatial position of the feature map $F\in {\mathbb{R}}^{C\times H\times W}$, where x,y∈[0,1] denote normalized pixel coordinates. To capture scale-dependent geometric patterns, these coordinates are expanded across *S* scales using a nonlinear mapping function:


$$\begin{array}{c} \Phi_{s}(x,y)=[\sin(2^{s}\pi x),\cos(2^{s}\pi x),\sin(2^{s}\pi y),\cos(2^{s}\pi y)],\;s=0,1,\ldots ,S-1. \end{array}$$

This mapping preserves both low-frequency structural cues (e.g., object location) and high-frequency details within the coordinate embedding space. Subsequently, all scale-specific encodings are concatenated and projected through a lightweight linear layer:


$$\begin{array}{c} E_{\text{coord}}=\sigma (W_{c}[\Phi_{0},\Phi_{1},\ldots ,\Phi_{S-1}]+b_{c}), \end{array}$$

where *W*_*c*_ and *b*_*c*_ are learnable parameters, and σ(·) denotes the activation function (ReLU).

Finally, the encoded coordinate tensor *E*_coord_ is adaptively fused with the intermediate visual feature *F* through a gating mechanism:


$$\begin{array}{c} F^{\prime }=\alpha \cdot F+(1-\alpha )\cdot (F⊙E_{\text{coord}}), \end{array}$$

where α is a learnable balancing coefficient and ⊙ represents element-wise multiplication. This adaptive fusion allows the model to selectively integrate geometric priors, guiding the detector toward regions with higher positional uncertainty—an essential property for accurately detecting small targets with limited pixel occupancy.

Overall, the MSCE enhances the spatial awareness of convolutional backbones by constructing a scale-adaptive coordinate representation that bridges implicit feature learning with explicit geometric reasoning, thereby providing the C2INR module with coordinate-informed guidance for fine-grained spatial alignment.

#### 3.2.2 INR feature enhancer (IFE).

Unlike conventional feature reweighting modules such as SE or FiLM, which primarily generate channel-wise scaling factors from globally pooled statistics, the proposed INR Feature Enhancer is motivated from a continuous function approximation perspective. Specifically, IFE models a coordinate-conditioned nonlinear mapping $\psi_{\theta }(\cdot )$ that treats spatial coordinates as continuous inputs to the modulation process. This formulation preserves the core property of classical implicit neural representations—learning a smooth coordinate-to-feature response field—rather than performing purely discrete feature recalibration. Consequently, the proposed design maintains the representational continuity of INR while remaining lightweight and fully compatible with CNN-based detection backbones.

Building upon this design principle, the IFE further integrates coordinate-aware information into the visual feature space to achieve continuous and resolution-independent feature refinement. It treats each pixel as a function of its coordinate embedding and corresponding visual features, enabling the network to reconstruct spatially continuous feature distributions beyond discrete convolutional sampling.

Given the intermediate visual feature $F^{\prime }\in {\mathbb{R}}^{C\times H\times W}$ obtained from the MSCE, the IFE first performs global average pooling to produce a compact descriptor $\bar{F}\in {\mathbb{R}}^{C}$. This descriptor is concatenated with the coordinate embedding *E*_coord_ to form a joint representation:


$$\begin{array}{c} z=[\bar{F}\;\|\;E_{\text{coord}}], \end{array}$$

where ‖ denotes feature concatenation along the channel dimension. This concatenated vector is then passed through a lightweight multi-layer perceptron (MLP) that models the implicit mapping from joint coordinate–visual space to enhanced feature space:


$$\begin{array}{c} E_{\text{INR}}=\psi_{\theta }(z)=\text{MLP}(z;\theta ), \end{array}$$

where $\psi_{\theta }$ denotes a learnable nonlinear function parameterized by θ. The output *E*_INR_ encodes both spatial continuity from coordinates and contextual semantics from visual features.

Subsequently, a residual modulation mechanism is applied to refine the original feature map:


$$\begin{array}{c} F_{\text{enh}}=F^{\prime }+\gamma \cdot (F^{\prime }⊙E_{\text{INR}}), \end{array}$$

where γ is a learnable scaling parameter that balances the influence of implicit enhancement, and ⊙ represents element-wise multiplication. This formulation ensures that INR-based refinement remains lightweight while maintaining gradient stability during training.

Through this implicit modulation, the IFE achieves adaptive alignment between coordinate and feature spaces, producing features that are more continuous, semantically consistent, andg resolution-robust. Such integration enables the detection head to better preserve fine-grained object boundaries and spatial coherence, which are critical for UAV small-object perception.

#### 3.2.3 Small object attention (SOA).

To further enhance the discriminability of tiny and densely distributed targets in UAV imagery, a Small Object Attention (SOA) module is introduced. Unlike conventional channel or spatial attention mechanisms that primarily emphasize global context, SOA is specifically designed to strengthen fine-grained responses of small objects by jointly modeling global context, local detail sensitivity, and high-frequency structural cues.

Given an input feature map $X\in {\mathbb{R}}^{B\times C\times H\times W}$, the SOA computes three complementary attention branches. To capture long-range semantic dependencies, global average pooling is first applied:


$$\begin{array}{c} g=\text{GAP}(X), \end{array}$$

followed by a lightweight bottleneck transformation:


$$\begin{array}{c} A_{g}=\sigma \left(W_{2}\;\delta (W_{1}g)\right), \end{array}$$

where $W_{1}\in {\mathbb{R}}^{\frac{C}{r}\times C}$ and $W_{2}\in {\mathbb{R}}^{C\times \frac{C}{r}}$ are learnable weights, *r* denotes the reduction ratio, δ(·) is the SiLU activation, and σ(·) is the sigmoid function. The resulting *A*_*g*_ models channel-wise global importance. To emphasize spatially fine structures that are critical for tiny object perception, SOA employs a point-wise nonlinear projection directly on the feature map:


$$\begin{array}{c} A_{l}=\sigma \left(W_{4}\;\delta (W_{3}X)\right), \end{array}$$

where *W*_3_ and *W*_4_ are implemented as 1 × 1 convolutions. This branch preserves spatial resolution while enhancing locally discriminative responses. Small objects often manifest as high-frequency patterns. To explicitly strengthen such cues, a depthwise separable convolution is applied:


$$\begin{array}{c} A_{d}=\sigma \left(W_{6}\left(W_{5}^{dw}(X)\right)\right), \end{array}$$

where $W_{5}^{dw}$ denotes a depthwise 3 × 3 convolution capturing high-frequency spatial variations, and *W*_6_ is a point-wise convolution for channel mixing. The three attention maps are averaged to obtain the final modulation weight:


$$\begin{array}{c} A=\frac{A_{g}+A_{l}+A_{d}}{3}, \end{array}$$

and the enhanced feature is computed via residual scaling:


$$\begin{array}{c} X_{\text{soa}}=X⊙A, \end{array}$$

where ⊙ denotes element-wise multiplication.

The proposed SOA is fundamentally different from conventional attention mechanisms in both design objective and feature modeling strategy. It is explicitly tailored for small-object perception by jointly leveraging global semantic context and high-frequency detail enhancement. The parallel multi-branch structure preserves computational efficiency while improving feature selectivity, and the depthwise enhancement pathway strengthens fine spatial discontinuities that are often suppressed in standard convolutional backbones. SOA serves as a lightweight yet effective module for amplifying weak small-object responses. When integrated with the coordinate-aware continuity modeling of C2INR, it further improves spatial discrimination capability and robustness in complex UAV scenes.

**Ethical Approval** No ethical clearance is required.

## 4 Experimental results

### 4.1 Experimental settings

#### 4.1.1 Datasets.

**AI-TOD** [18] is a benchmark for tiny object detection in aerial imagery, containing 28,036 images with over 700,000 instances. Its average object size is only 12.8 pixels, making it highly challenging for detectors. **UAVDT** [19] is a large-scale dataset of 50,000 UAV frames annotated with three vehicle categories. Challenges stem from small object sizes and diverse weather and lighting conditions, making it suitable for robustness evaluation. **VisDrone** [20] contains 10,209 images with over 540,000 instances across 10 categories. It is characterized by dense scenes, severe occlusions, and large scale variations, providing a rigorous benchmark for crowded UAV scenarios. **PASCAL VOC** [21] are canonical benchmarks for generic object detection, containing 20 everyday object categories and approximately 16,000 images in total. Although the objects in VOC are significantly larger and less crowded than those in UAV datasets, we include VOC for generalization evaluation. Testing on VOC allows us to assess whether the proposed C2INR framework—originally designed for small-object UAV detection—can transfer its benefits to broader detection tasks without domain-specific tuning. This cross-domain setting provides a rigorous measure of the model’s stability and adaptability beyond aerial imagery.

### 4.2 Implementation details

The experiments are conducted on a single NVIDIA RTX 3090 GPU with 24 GB of memory. Our algorithm is implemented using the PyTorch framework, and the optimizer is SGD. The initial learning rate is set to 0.01. During preprocessing, the input size of the model is set to 640×640, and the batch size is 16. The training is performed for 100 epochs.

### 4.3 Main results

#### 4.3.1 AI-TOD dataset results.

Table 1 presents the results on the AI-TOD benchmark, which contains extremely small and densely packed objects. The proposed method achieves 55.4% mAP50, 19.5% mAP75, and 25.2% mAP50:95, outperforming most existing CNN-based and transformer-based detectors. Compared with YOLOv8-M, our method delivers consistent improvements of +2.9%, + 3.0%, and +2.3% across the three metrics, demonstrating that coordinate-aware implicit modeling enhances localization precision, particularly under stricter IoU thresholds. The method also surpasses specialized small-object detectors, such as BRSTD and ESL-YOLO, while maintaining a compact CSPDarkNet53 backbone. To further verify backbone generalization, additional experiments with ResNet18 and ResNet50 are reported. Although lighter backbones yield slightly lower accuracy due to reduced representational capacity, the proposed framework consistently improves performance across all backbones. CSPDarkNet53 achieves the best results, indicating that its cross-stage partial aggregation better complements the proposed coordinate-aware modeling for extremely small object scenarios.

**Table 1:** Performance comparison of different detectors on the AI-TOD dataset. “-” indicates that the corresponding values were not reported in the original literature.

Detectors	Backbone	mAP50(%)	mAP75(%)	mAP50:95(%)	Param(M)
FoveaBox	ResNet50	19.8	5.1	8.1	–
Double Head R-CNN*	ResNet50	24.3	6.7	10.1	–
RetinaNet	ResNet50	24.2	4.6	8.9	–
Faster-RCNN	ResNet50	29.9	9.4	12.8	–
ATSS	ResNet50	30.6	8.5	12.8	–
DETR-DC5*	ResNet50	32.5	3.9	10.4	–
Cascade R-CNN	ResNet50	34.2	11.2	15.1	–
DetectoRS	ResNet50	35.5	12.5	16.1	–
DotD	ResNet50	51.4	12.3	20.4	–
Deformable-DETR*	ResNet50	50.0	10.5	48.9	–
YOLOv8-M	CSPDarkNet53	52.5	16.5	22.9	25.8
HANet	ResNet50	53.7	14.4	22.1	26.4
DINO-Deformable-DETR*	ResNet50	56.6	15.4	23.2	44.0
KLDet	–	–	–	19.6	32.0
MENet-Swin-T*	Swin-T	56.2	15.0	23.2	41.9
ORFENet	–	55.4	18.2	24.8	32.7
BRSTD	CSPDarkNet53	54.2	16.6	23.6	–
FSANet	ResNet50	48.1	14.0	20.3	31.9
NWD-RKA	ResNet50	53.5	16.8	23.4	540.1
RFLA	ResNet50	55.2	18.5	24.8	–
DCN-YOLO	CSPDarkNet53	56.6	15.4	23.9	–
ESL-YOLO	CSPDarkNet53	51.8	–	23.5	11.96
QueryDet	ResNet50	54.3	–	–	18.93
ADAS-GPM	ResNet50	49.7	12.4	20.1	–
MAV23	Swin-T	47.7	8.0	17.2	–
Ours	ResNet18	52.1	17.6	23.1	15.21
Ours	ResNet50	54.0	18.7	24.4	41.27
Ours	CSPDarkNet53	55.4	19.5	25.2	34.86

#### 4.3.2 UAVDT dataset results.

As shown in Table 2, the proposed method achieves 39.2% mAP50, 29.1% mAP75, and 25.3% mAP50:95, achieving state-of-the-art performance among lightweight YOLO-based detectors. Relative to YOLOv8 (15.7 mAP50:95), C2INR obtains a substantial gain of +9.6%, highlighting the effectiveness of continuous coordinate modeling in dynamic UAV scenes with motion blur and rapid viewpoint changes. The improvements over CSSDet, PP-YOLOE, and heavier detectors such as PETNet and RemDet-L further demonstrate the robustness of the proposed approach. The significant margin in mAP75 confirms that MSCE and IFE enhance high-IoU localization stability, which is critical for fast-moving UAV platforms. The cross-backbone comparison shows similar trends: performance steadily improves from ResNet18 to ResNet50 and further to CSPDarkNet53. Importantly, even with the lightweight ResNet18 backbone, the proposed modules maintain competitive accuracy, verifying that the gains are not backbone-specific but stem from the proposed C2INR design.

**Table 2:** Performance comparison of different detectors on the UAVDT dataset. “-” indicates that the corresponding values were not reported in the original literature.

Detectors	Backbone	mAP50(%)	mAP75(%)	mAP50:95(%)	Param(M)
ClusDet	ResNet50	26.5	12.5	13.7	–
DMNet	ResNet50	24.6	16.3	14.7	–
CDMDet	ResNet50	35.5	22.4	20.7	–
GLSAN	ResNet50	30.5	21.7	19.0	–
UCGNet	CSPDarkNet53	36.7	18.0	19.1	–
ADaZoom	ResNet50	33.6	21.3	19.6	–
CZDet	ResNet50	34.1	21.3	19.8	–
QueryDet	ResNet50	27.2	16.6	14.3	–
SDPDet	ResNet50	32.0	23.1	20.0	–
YOLO-World	CSPDarkNet53	35.8	–	–	–
YOLOv8	CSPDarkNet53	29.1	15.3	15.7	43.63
CSSDet	CSPDarkNet53	34.9	24.8	21.7	57.99
RTMDet	CSPDarkNeXt	33.0	21.8	19.9	52.26
DPH-YOLOv8	CSPDarkNet53	33.4	–	20.3	–
PPYOLOE	PPYOLOECSPDarkNet53	33.1	22.7	20.4	51.35
YOLOX	CSPDarkNet53	25.5	16.9	15.4	54.16
Groundin-DINO	Swin-T	36.0	–	–	–
YOLC	HRNet	30.9	20.1	19.3	–
RemDet-L*	–	34.5	22.1	20.6	–
CEASC	–	30.9	17.8	17.1	–
AMRNet	–	30.4	19.8	18.2	–
Efficient-YOLOv7-Drone	Efficient	36.0	20.1	20.0	–
PETNet	–	38.6	22.3	21.5	–
ACDet	ResNet18	31.8	18.8	18.0	–
Ours	ResNet18	36.8	25.7	23.6	15.21
Ours	ResNet50	38.5	27.6	24.6	41.27
Ours	CSPDarkNet53	39.2	29.1	25.3	34.86

#### 4.3.3 VisDrone dataset results.

Table 3 shows that our method achieves 43.6% mAP50, 26.8% mAP75, and 26.4% mAP50:95, outperforming many one-stage and two-stage detectors. Compared with YOLOv8-M, C2INR improves mAP50 and mAP50:95 by +1.2%, and surpasses variants such as FFCA-YOLO and Mamba-YOLO, confirming the effectiveness of coordinate-aware implicit modeling. Moreover, the proposed method reaches accuracy comparable to transformer-based detectors while maintaining lower computational cost. The backbone ablation further demonstrates strong generalization. While CSPDarkNet53 provides the highest accuracy due to richer multi-scale features, the consistent gains observed on ResNet18 and ResNet50 confirm that the proposed modules are backbone-agnostic. Overall, these results validate the robustness and cross-architecture effectiveness of C2INR across datasets with diverse object densities and environmental conditions.

**Table 3:** Performance comparison of different detectors on the VisDrone dataset. “-” indicates that the corresponding values were not reported in the original literature.

Detectors	Backbone	mAP50(%)	mAP75(%)	mAP50:95(%)	Param(M)
SSD	VGG16	30.22	13.15	11.74	–
RetinaNet	ResNet50	21.2	–	–	19.83
Faster-RCNN	ResNet50	43.6	25.9	25.5	–
Faster-RCNN	VGG16	30.79	13.52	12.8	–
MaskRCNN	ResNet50	32.04	15.24	15.47	–
Cascade R-CNN	ResNet50	33.5	17.32	18.33	–
Faster-RCNN+FPN	ResNet50	47.32	24.33	26.01	–
YOLOv5M	CSPDarkNet53	29.5	–	15.6	20.9
YOLOv5l	CSPDarkNet53	34.3	–	18.6	46.16
YOLOv7	CSPDarkNet53	34.1	–	18.1	9.34
YOLO-HV	NextVIT	38.1	–	19.9	38.53
YOLO12	CSPDarkNet53	42.0	–	25.1	26.4
YOLOv8M	CSPDarkNet53	41.8	–	25.2	25.8
SR-YOLO	CSPDarkNet53	41.6	–	23.9	7.61
YOLOv9C	CSPDarkNeXt	44.8	–	–	25.51
QueryDet	CSPDarkNet53	31.6	–	–	18.91
Deformable-DETR	ResNet50	–	–	–	39.84
ATSS	ResNet50	31.7	–	18.6	–
DTSSNet	MobileNetV2	41.1	26.9	25.5	10.1
TPH-YOLOv5	–	36.3	–	19.4	–
FFCA-YOLO	CSPDarkNet53	41.1	–	22.5	–
YOLO13	CSPDarkNet53	40.5	–	24.2	27.6
DetectoRS*	ResNet50	40.1	22.3	22.3	–
RT-DETR	ResNet18	35.9	–	–	20.0
Mamba-YOLO-B	CSPDarkNet53	40.8	–	23.9	21.8
Ours	ResNet18	41.5	24.9	24.3	15.21
Ours	ResNet50	42.8	26.0	25.5	41.27
Ours	CSPDarkNet53	43.6	26.8	26.4	34.86

#### 4.3.4 Qualitative results.

**Fig 3:**
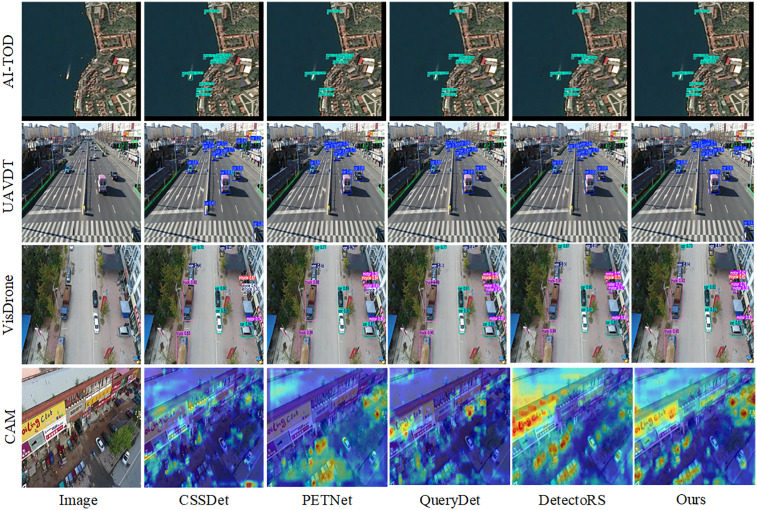
Visualization of heatmaps and detection results for C2INR. Brighter areas in the heatmaps indicate stronger model attention. Detection results produced by the proposed model on representative images from the publicly available VisDrone, UAVDT, and AI-TOD datasets. The original images are sampled from the corresponding datasets and annotated with predicted bounding boxes for visualization. Dataset images are reproduced from their original publications under CC BY licenses (Zhu et al., 2018; Du et al., 2018; Wang et al., 2021).

**Fig 4:**
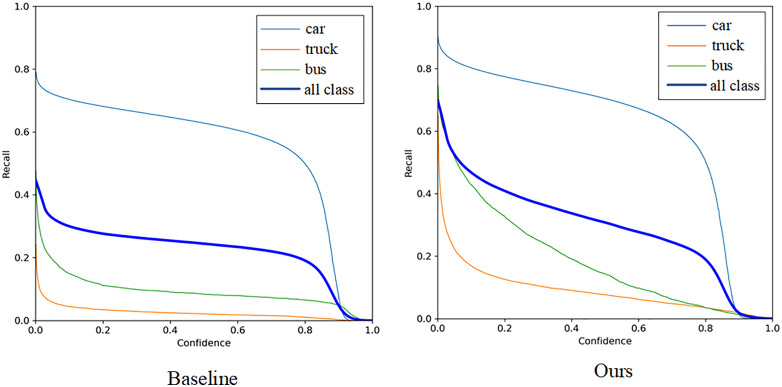
Recall–Confidence curves of the baseline and the proposed method across different vehicle categories and the overall performance.

**Fig 5:**
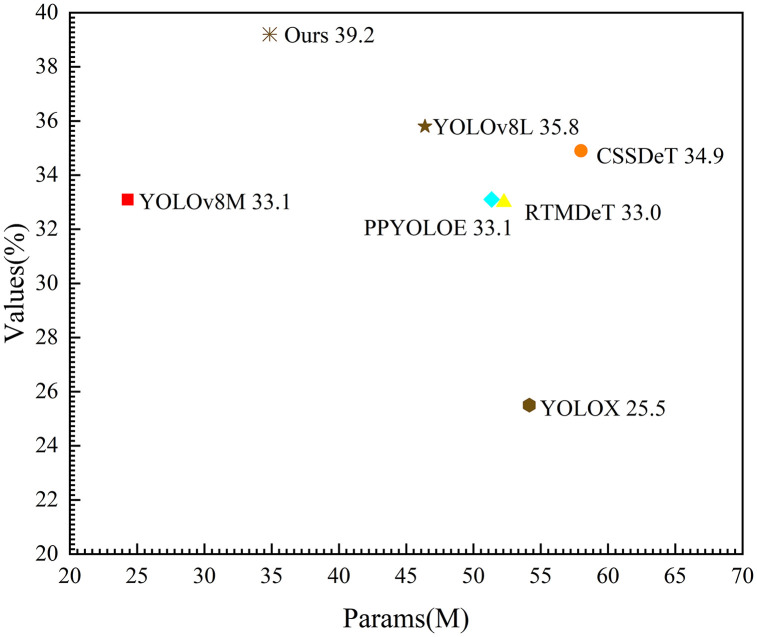
Comparison of the proposed method with different models on the UAVDT dataset.

Figs 3-5 jointly demonstrate thes qualitative advantages and overall effectiveness of the proposed C2INR framework. As shown in Fig 3, C2INR yields clearer and more accurate detections across AI-TOD, UAVDT, and VisDrone. Compared with CSSDet, PETNet, QueryDet, and DetectoRS, our method produces tighter bounding boxes and reliably detects small, distant, or low-contrast objects that competing algorithms frequently miss or localize imprecisely. The CAM visualizations reveal that C2INR generates sharper and more concentrated activation responses around true object regions while suppressing background interference, confirming that the MSCE and IFE components effectively enhance geometry-aware and spatially coherent feature learning.

Fig 4 Further validates these observations. Across a wide confidence range, the proposed method consistently achieves higher precision than the baseline, with particularly pronounced improvements for challenging categories such as trucks and buses. The smoother Precision–Confidence curves and the elevated high-confidence precision demonstrate improved robustness, more reliable confidence calibration, and reduced prediction ambiguity, reflecting C2INR’s stronger localization stability under varying imaging conditions.

Fig 5 Compares model performance with respect to parameter complexity on the UAVDT dataset. Notably, C2INR achieves 39.2% mAP50 while maintaining a moderate parameter count, outperforming popular detectors such as YOLOv8-M, YOLOv8-L, PP-YOLOE, RTMDet, CSSDet, and YOLOX across both accuracy and efficiency dimensions. This favorable trade-off indicates that the introduction of coordinate-aware implicit modeling significantly boosts small-object perception without incurring substantial computational overhead.

Overall, these qualitative and comparative results demonstrate that C2INR effectively enhances spatial consistency, strengthens fine-grained attention, and achieves superior accuracy–efficiency balance for UAV-based small-object detection.

### 4.4 Ablation study

**Fig 6:**
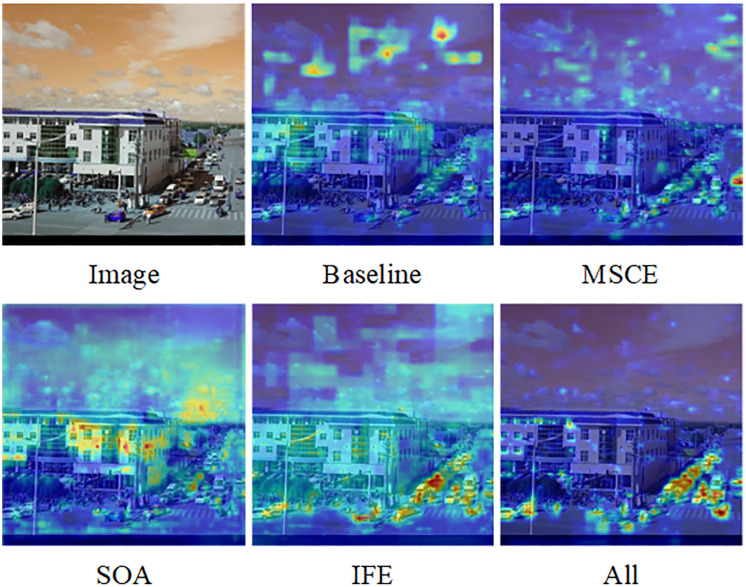
Heatmaps with tshe MSCE, SOA, and IFE modules. Example images are sampled from the publicly available AI-TOD dataset. Reprinted from Wang et al., “AI-TOD: A Large-Scale Dataset for Tiny Object Detection in Aerial Images,” under a CC BY license. Original copyright 2021.

**Fig 7:**
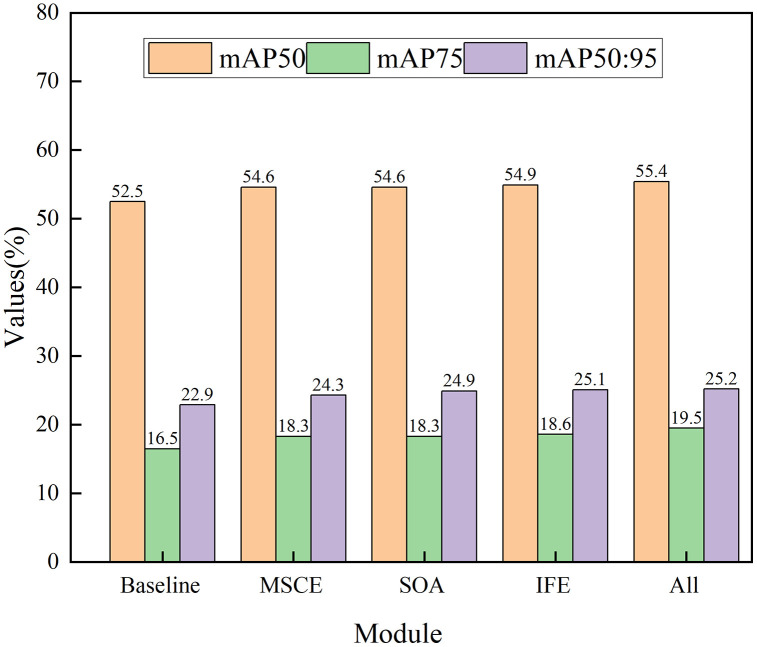
Comparison of the MSCE, SOA, and IFE modules and baseline on the AI-TOD dataset.

The ablation results in Table 4, together with the visualizations in Figs 6 and 7, collectively demonstrate the effectiveness and complementarity of the proposed MSCE, SOA, and IFE modules. Introducing MSCE yields clear improvements across all evaluation metrics, indicating its ability to enrich geometric priors and strengthen spatial continuity for small-object localization. SOA provides comparable gains by enhancing multi-path attention to suppress background noise and emphasize fine details in dense UAV scenes. IFE delivers the largest single-module improvement in mAP50:95, confirming that coordinate-conditioned implicit refinement substantially enhances feature discrimination. When integrated, the complete C2INR module achieves the best ablation performance, reflecting the strong complementarity among the three components. These improvements translate consistently to the overall benchmark results. As shown in Table 5, the proposed method attains the best detection performance on the UAVDT dataset, reaching 39.2% mAP50 and 25.3% mAP50:95, significantly outperforming all competing detectors. Meanwhile, the model remains computationally efficient, containing only 34.86M parameters and requiring 96.8 GFLOPs, both lower than those of the strong baselines. Notably, despite the accuracy gains, the proposed method preserves real-time capability at 118.4 FPS, surpassing most existing detectors. The heatmap comparisons further corroborate these findings: MSCE improves spatial coherence, SOA enhances focus on small and low-contrast regions, and IFE sharpens structural cues, while their combination produces the most precise and concentrated activations around target objects. Overall, these results verify that C2INR strengthens spatial fidelity and detection robustness while introducing minimal computational overhead, demonstrating its practicality for real-time UAV small-object detection.

**Table 4:** Ablation study on the AI-TOD dataset evaluating the individual and combined effects of the MSCE,SOA and IFE modules.

MSCE	SOA	IFE	mAP50(%)	mAP75(%)	mAP50:95(%)
×	×	×	52.5	16.5	22.9
✓	×	×	54.6	18.3	24.3
×	✓	×	54.5	18.1	24.9
×	×	✓	54.9	18.6	25.1
✓	✓	✓	55.4	19.5	25.2

**Table 5:** Quantitative comparison of detection accuracy and computational efficiency.

Model	mAP50(%)	Param(M)	GFLOPs	FPS
Baseline	29.1	43.63	109.5	112.0
YOLOX	25.5	54.16	121.3	98.5
PPYOLOE	33.1	51.35	118.7	92.3
CSSDet	34.9	57.99	132.6	85.6
RTMDet	33.0	52.26	115.2	96.1
Ours	39.2	34.86	96.8	118.4

### 4.5 Parameter sensitivity analysis of the C2INR components

#### 4.5.1 Effect of coordinate encoding dimension (MSCE).

**Fig 8:**
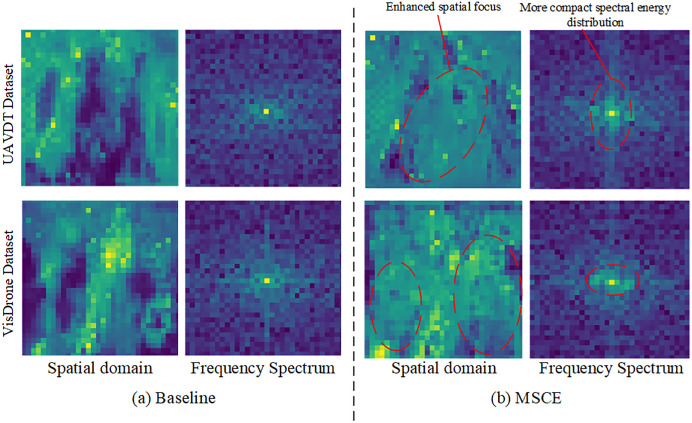
Visualization of spatial and frequency responses produced by the MSCE module on UAVDT and VisDrone datasets. The first and third columns show spatial-domain activation maps of the baseline and MSCE, respectively, while the second and fourth columns present the corresponding frequency spectra. Compared with the baseline, MSCE yields more concentrated spatial responses over object regions and a more compact high-frequency energy distribution, indicating enhanced spatial continuity modeling and reduced background interference. The improvement is consistently observed on both datasets, demonstrating the effectiveness and generalization capability of MSCE for UAV small-object detection.

Table 6 shows that increasing the coordinate encoding dimension leads to consistent performance gains, particularly at higher IoU thresholds. Specifically, enlarging the embedding dimension from 32 to 192 improves mAP50:95 from 24.8% to 26.4%, demonstrating that richer multi-frequency coordinate representations provide more expressive spatial priors for the detector. As illustrated in Fig 8, the proposed MSCE produces more spatially concentrated responses over object regions and a more compact high-frequency energy distribution compared with the baseline encoder. This phenomenon suggests that MSCE effectively enhances the discriminability of object-related features while suppressing background noise and clutter. By leveraging multi-scale coordinate encoding, the module improves spatial continuity modeling and reduces the aliasing effect commonly observed in conventional CNN feature maps. the steady improvement in mAP75 further confirms that MSCE enhances localization precision by strengthening the INR-based spatial feature fusion process. The improved high-IoU performance indicates that the detector benefits not only from better object awareness but also from more accurate boundary delineation. This advantage is particularly important for UAV small-object detection, where objects occupy only a few pixels and are easily overwhelmed by complex backgrounds. Overall, these results validate that increasing the coordinate encoding capacity in MSCE leads to more robust spatial representation learning and more precise small-object localization.

**Table 6:** Effect on the coord encoding dim of C2INR module on the VisDrone dataset.

coord encoding dim	mAP50(%)	mAP75(%)	mAP50:95(%)
32	42.1	25.1	24.8
64	42.5	25.4	25.1
128	42.8	25.8	25.7
192	43.6	26.8	26.4

#### 4.5.2 Effect of minimum spatial attention radius (MSAR).

As shown in Table 7, MSAR exhibits a non-linear influence on detection accuracy. Very small radii yield the highest performance (40.8% mAP50), suggesting that fine-grained attention is essential for highlighting small or fast-moving objects in UAVDT. However, overly small radii may increase noise sensitivity, while larger radii such as 16–32 oversmooth spatial details and weaken cues critical to small-object detection. The best balance is achieved at MSAR = 2, indicating that SOA benefits most from a compact spatial receptive field tailored to high-density UAV scenes.

**Table 7:** Effect on the minimum spatial attention radius (MSAR) of SOA on the UAVDT dataset.

MSAR	mAP50(%)	mAP75(%)	mAP50:95(%)
2	40.8	30.5	26.2
4	39.9	30.2	25.9
8	39.2	29.1	25.3
16	38.4	27.7	24.2
32	40.0	30.2	26.1

#### 4.5.3 Effect of IFE Hidden dimension.

Table 8 analyzes the hidden dimensionality of the MLP in the IFE module. Moderate hidden sizes (16–32) achieve the strongest overall results, whereas larger dimensions (64–128) result in diminishing or negative returns. This pattern suggests that coordinate-conditioned refinement does not require large-capacity transformations; instead, lightweight modulation is sufficient to strengthen spatial–semantic interactions without introducing redundancy. These findings highlight the efficiency of the proposed IFE design, demonstrating that compact representations yield the most stable improvements in UAV detection accuracy.

**Table 8:** Effect on the hidden dim of IFE on the UAVDT dataset.

hidden dim	mAP50(%)	mAP75(%)	mAP50:95(%)
16	40.0	30.1	25.9
32	39.9	29.1	25.5
64	39.2	29.1	25.3
128	39.1	29.6	25.5

### 4.6 Impact of module quantity

**Fig 9:**
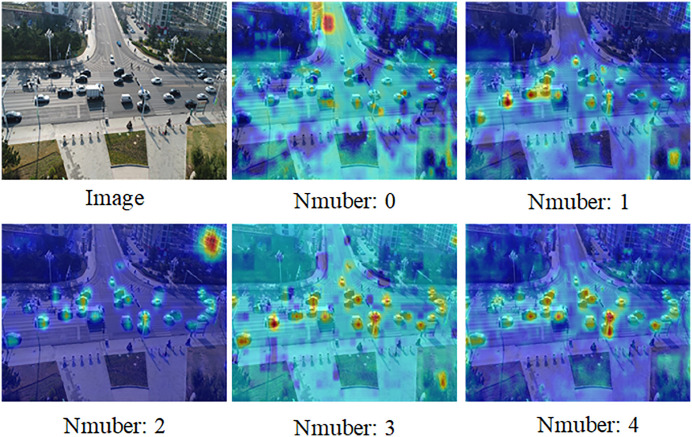
Visualization of feature activation maps under different numbers of C2INR. Example frame from the UAVDT dataset. Reprinted from Du et al., The Unmanned Aerial Vehicle Benchmark: Object Detection and Tracking, under a CC BY license, with permission from the authors. Original copyright 2018.

Table 9 and Fig 9 examine the influence of stacking multiple C2INR modules on detection accuracy and feature activation behavior. As shown in Table 9, introducing even a single C2INR module leads to a marked improvement over the baseline, confirming the effectiveness of integrating coordinate-aware implicit modeling. Performance continues to rise as additional modules are incorporated, and the best results are achieved when four C2INR blocks are employed. The consistent increase in mAP75 indicates enhanced high-IoU localization, suggesting that deeper C2INR integration strengthens spatial continuity and structural fidelity.

**Table 9:** Effect on the numbers of C2INR.

Number	mAP50(%)	mAP75(%)	mAP50:95(%)
0	33.1	22.1	20.2
1	37.5	26.6	23.5
2	38.4	27.2	23.8
3	39.0	27.4	24.1
4	39.2	29.1	25.3

The activation maps in Fig 9 corroborate these quantitative trends. With one C2INR block, the model already generates more coherent object responses than the baseline. As the number of modules increases, the activations become progressively more concentrated and less noisy, revealing clearer boundaries between foreground targets and background regions. When four modules are used, the feature maps exhibit the most pronounced and well-localized responses, indicating improved geometric reasoning and greater sensitivity to small, densely distributed UAV objects. these results show that stacking C2INR modules systematically enhances fine-grained spatial representation, leading to both measurable performance gains and progressively sharper, more discriminative feature activation patterns.

## 5 Generalization validation

The generalization results on the VOC dataset in Table 10 indicate that the proposed C2INR module transfers effectively beyond UAV scenarios. Compared with the YOLOv8 baseline, our method achieves notable gains in Precision, Recall and mAP50, demonstrating that the coordinate-aware representations produced by MSCE and IFE provide stable improvements even in datasets with larger objects and more diverse scenes. Across a wide range of detectors—including two-stage models (Faster R-CNN, DDOD), anchor-free frameworks (YOLOF, YOLOX), and transformer-based detectors (DDQ, DINO)—the proposed method achieves the highest overall mAP50 while maintaining a balanced precision–recall profile. These results confirm that C2INR not only enhances small-object detection in UAV imagery but also offers strong cross-domain robustness, highlighting its practical applicability in broader detection tasks.

**Table 10:** Generalization performance of C2INR module on the VOC dataset.

Model	Precision(%)	Recall(%)	mAP50(%)
YOLOv8	71.3	66.2	71.3
Faster R-CNN	60.5	62.0	65.5
Cascade R-CNN	65.4	57.4	62.2
FreeAnchor	15.9	26.4	43.0
YOLOF	64.0	63.8	69.6
YOLOX	70.9	36.5	56.0
VarifocalNet	63.7	61.6	75.2
DDOD	76.8	56.3	73.4
RTMDet	51.9	61.1	73.2
DAB-DETR	–	58.9	72.2
DDQ	–	59.9	73.3
DINO	72.5	57.3	70.6
Ours	78.3	69.9	76.7

## 6 Discussion

The results show that incorporating coordinate-aware implicit modeling into CNN-based detectors provides clear advantages for small-object detection. By introducing multi-frequency coordinate embeddings and coordinate-conditioned modulation, C2INR enhances spatial continuity and sub-pixel localization, addressing limitations of purely discrete convolutional features. Although the additional computation is modest, further efficiency gains may be achieved through lighter positional encodings or frequency-selection strategies.

Performance across multiple datasets suggests good generalizability, yet the behavior of INR under strong domain shifts—such as infrared or low-light UAV imagery—remains an open area for study. Future work may explore domain-invariant coordinate representations and integration with hybrid architectures such as transformers or dynamic filtering networks.

Overall, the study highlights coordinate-aware implicit representation as a compact and effective enhancement to existing detectors, providing a promising direction for designing lightweight and spatially precise models for small-object perception.

## 7 Conclusion

This study introduces a coordinate-aware implicit representation framework designed to enhance spatial continuity, geometric fidelity, and localization accuracy in UAV small-object detection. By integrating multi-frequency coordinate embeddings through MSCE and implicit feature refinement through IFE, the proposed C2INR module bridges conventional discrete convolution with continuous coordinate-conditioned modeling. This unified design enables the detector to better capture fine-grained spatial structures and maintain coherent representations across scales.

Comprehensive experiments on AI-TOD, UAVDT, and VisDrone demonstrate that C2INR yields consistent improvements over strong CNN- and transformer-based detectors, particularly under strict IoU criteria. The ablation and sensitivity studies further highlight the complementary contributions of MSCE, SOA, and IFE in enhancing geometric awareness and discriminative capability. Moreover, the generalization results on PASCAL VOC verify that the proposed module extends effectively beyond UAV imagery, indicating promising applicability in broader object detection tasks involving small, sparse, or scale-varying targets.

In summary, C2INR provides a principled and efficient approach to integrating coordinate-driven continuity into modern detection frameworks. Future work will investigate dynamic implicit fields, adaptive coordinate priors, and lightweight formulations to further improve robustness. Extending the INR paradigm to multi-modal UAV sensing, such as RGBT, SAR, and LiDAR, also represents a valuable direction for enhancing detection under challenging environmental or cross-domain scenarios.

## Supporting information

S1 File Supplementary material includes implementation details and training settings. Source code: https://github.com/wtc0214/INR.
(DOC)
