# Supplementary material for: Coordinate aware implicit neural representation for UAV small object detection
Source: PLoS One. 2026 Jun 10;21(6):e0350990. doi: 10.1371/journal.pone.0350990 (PMC13252807; doi:10.1371/journal.pone.0350990)
Supplement: S1 File — Source code: https://github.com/wtc0214/INR. (DOC) [file pone.0350990.s001.doc]

***Supporting Information***

***S1 File. Supplementary material provides implementation details, dataset configuration, and training settings. Source code and additional resources are available at:*** [***https://github.com/wtc0214/INR***](https://github.com/wtc0214/INR)
